# Pulse Width Modulation Applied to Olfactory Stimulation for Intensity Tuning

**DOI:** 10.1371/journal.pone.0145373

**Published:** 2015-12-28

**Authors:** Patrice Andrieu, Pierre-Édouard Billot, Jean-Louis Millot, Tijani Gharbi

**Affiliations:** 1 EA 481: Laboratory of Integrative and Clinical Neuroscience, University of Franche-Comté, Besançon, France; 2 EA 4662: Nanomedicine Imagery and Therapeutics Laboratory, University of Franche-Comté, Besançon, France; Université Lyon, FRANCE

## Abstract

For most olfactometers described in the literature, adjusting olfactory stimulation intensity involves modifying the dilution of the odorant in a neutral solution (water, mineral, oil, etc.), the dilution of the odorant air in neutral airflow, or the surface of the odorant in contact with airflow. But, for most of these above-mentioned devices, manual intervention is necessary for adjusting concentration. We present in this article a method of controlling odorant concentration via a computer which can be implemented on even the most dynamic olfactometers. We used Pulse Width Modulation (PWM), a technique commonly used in electronic or electrical engineering, and we have applied it to odor delivery. PWM, when applied to odor delivery, comprises an alternative presentation of odorant air and clean air at a high frequency. The cycle period (odor presentation and rest) is 200 ms. In order to modify odorant concentration, the ratio between the odorant period and clean air presentation during a cycle is modified. This ratio is named duty cycle. Gas chromatography measurements show that this method offers a range of mixing factors from 33% to 100% (continuous presentation of odor). Proof of principle is provided via a psychophysical experiment. Three odors (isoamyl acetate, butanol and pyridine) were presented to twenty subjects. Each odor was delivered three times with five values of duty cycles. After each stimulation, the subjects were asked to estimate the intensity of the stimulus on a 10 point scale, ranging from 0 (undetectable) to 9 (very strong). Results show a main effect of the duty cycles on the intensity ratings for all tested odors.

## Introduction

One of the difficulties often encountered in the design of olfactometers is the concentration setting. Zwaardemaker, who published one of the first olfactometers [1], proposed changing the evaporation surface in order to adjust odor concentration. This principle was used by other research teams, but with different implementations [2]. Another method consists in diluting odorant airflow in clean air. Kobal and Bozza [3, 4] proposed to saturate air with odorous vapor before the air dilution: this provides the experimenter with knowledge of the concentration without requiring any measurement. The adjustment of concentration can also be obtained by diluting the liquid odorant in a neutral solution (water, mineral oil or diethyl phthalate) [5, 6]. Lorig introduced the association of the couple of last methods described above [7], a device from which several olfactometers were inspired [8–11].

It is now well known that odorant concentration modulates psychophysical ratings of intensity in human subjects, whether with liquid dilutions in solvents [12] or with air dilutions [13]. More recently, Noam Sobel and his colleagues have shown that the perceived intensity seems to be correlated with neural activity within brain regions involved in olfactory processing, like the amygdala [14]. A quick and accurate control of concentration (and thus, of intensity) is crucial in olfactory studies.

For most devices, adjusting concentration needs manual intervention. An automatization of this process was proposed by Eyferth [15] but its construction and implementation are complex and expensive. The air dilution method can also be improved by using two mass flow controllers, an efficient but expensive solution which can be difficult to implement. Thus, we describe in this manuscript an alternative method enabling the control of the concentration thanks to a computer: Pulse Width Modulation (PWM).

**Fig 1:**
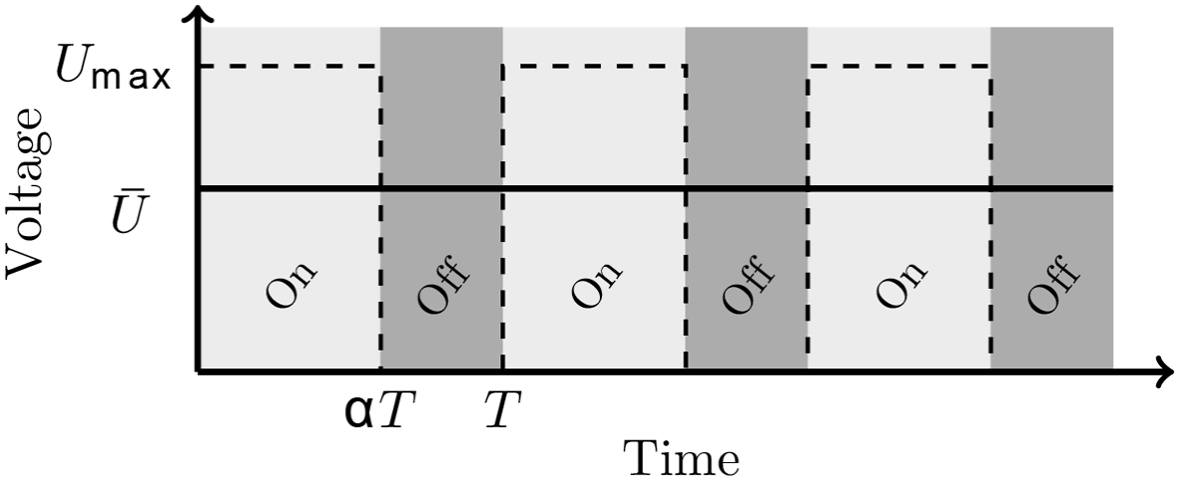
Principle of PWM. The voltage $\bar{U}=\alpha .U_{max}$ is obtained by interrupting the tension *U*_*max*_ every T period during *α*T.

PWM is commonly used in electronic or electrical engineering. It allows the control of the power supplied to electrical devices. Power-on and power-off phases are alternated at high switching frequency. The duty cycle, commonly noted as *α*, is a ratio between the period of “power-on” and “power-off” phase (Fig 1). The duty cycle and average of voltage (and current) are linked by the relation $\bar{U}=\alpha .U_{max}$.

PWM, when applied to odor delivery, comprises an alternative presentation of odorant air and clean air at a high frequency. It can be implemented with most olfactometers without major transformation.

The first step is to determine the range of mixing factors thanks to gas chromatography measurements. In a second step, we measure the effect of these mixing factors on intensity ratings in human subjects in order to assess the applicability of the device.

## Materials and Methods

### Overall presentation of olfactometer

**Fig 2:**
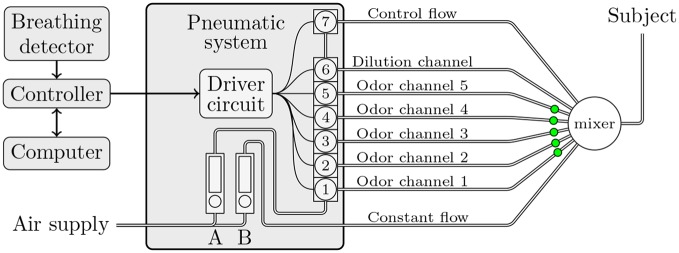
Schematic view of olfactometer. This “Lorig design” was modified by adding a dilution channel enabling its use with PWM.

With “Lorig design” olfactometers [7], it is possible to control the concentration with a computer thanks to PWM. In our case, we used an olfactometer which we already described in a previous publication [11]. Therefore we will only briefly describe the functioning. This olfactometer consists of three interconnected subsystems: (1) the controller which is an interface between the computer and the Pneumatic System (PS), (2) the breathing detector which synchronizes the controller with the subject’s respiration if necessary, and (3) the PS which regulates airflow and directs it to the subject via the odor channel or directly to the mixer (Fig 2).

### Pulse Width Modulation and odor delivery

**Fig 3:**
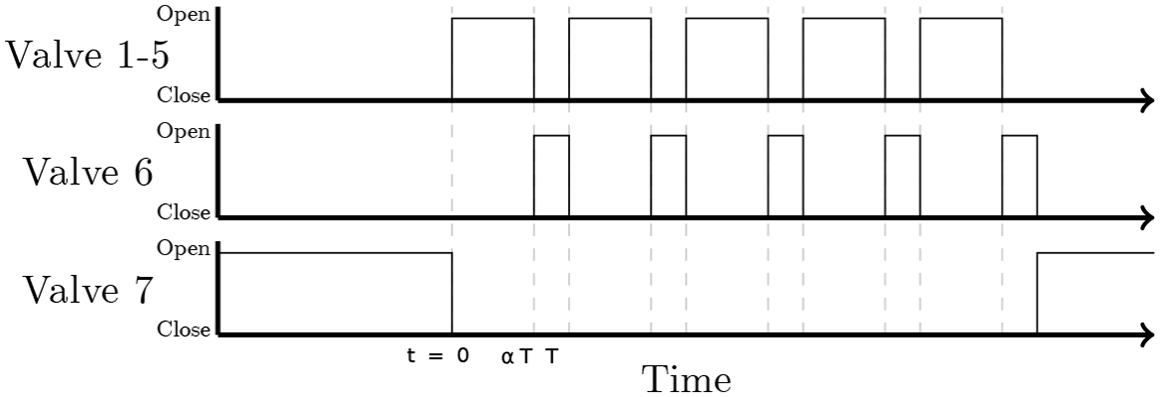
Chronogram of the activation of valves. During the rest period, valve 7 delivers clean air. During the stimulation period, the odor pulse is delivered through valve 1–5 every T period and the flow is compensated with valve 6.

To use the principle of the PWM with the olfactometer which is already described in our previous article, we needed to change the connection between the PS and the mixer and reprogram the controller. We would recommend consulting Fig 2 while reading this section. The PS is comprised of two flowmeters: A manages the airflow which supplies the solenoid valves, B manages the constant airflow. The controller drives the valves via a driver circuit which prolongs valve life and limits power consumption. Valves 1 to 6 are six normally closed manifold valves/selectors. For delivering odor with the PWM method, the controller quickly switches between the valve corresponding to an odor channel (valves 1 to 5) and valve 6 corresponding to the dilution channel. The period of switching noted *T* is 200 ms which corresponds to 5 Hz. When no odor is delivered, the normally open valve 7, which manages the control flow, is open to keep a constant flow at the mixer outlet (Fig 3).

### Theoretical approach

**Fig 4:**
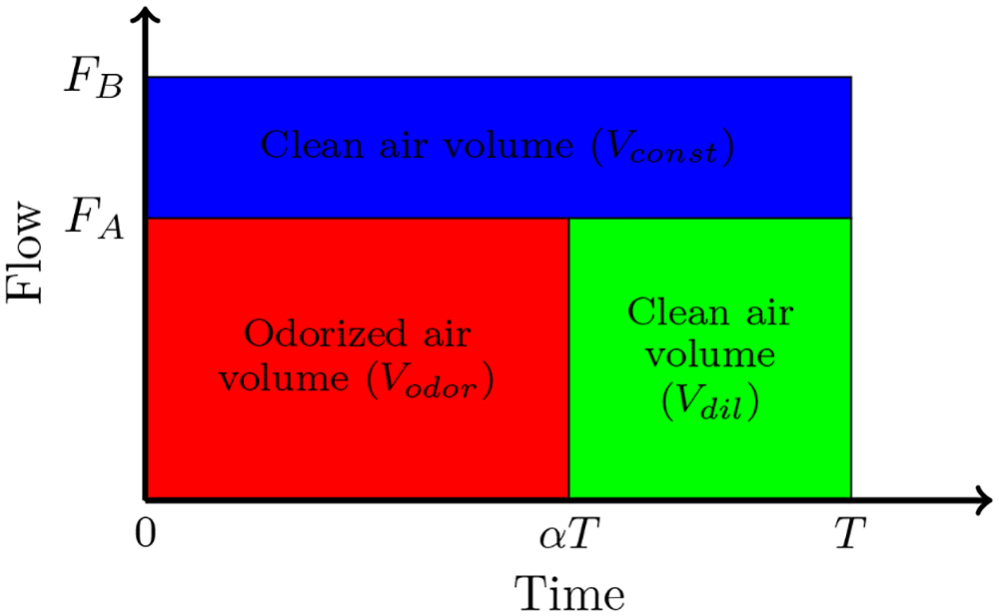
Representation of the volumes delivered during a *T* period. *F*_*A*_ and *F*_*B*_ correspond to the airflow managed by flowmeters A and B (Fig 2) respectively. In blue, the air volume due to the constant flow. In red and green, the volume of odor pulse and the volume required to keep a constant flow.

To determine the relationship between the concentration and the duty cycle, we propose the simplified model described in Fig 4.

In this model we do not take into account the slow rate of the PS. The relationship between the final concentration (at the mixer outlet) *C*_*final*_ and the concentration in airflow crossing the odor diffuser *C*_*Vodor*_ is:

$$\begin{array}{ccc} C_{final} & = & C_{Vodor}\frac{V_{odor}}{V_{total}}\\ C_{final} & = & C_{Vodor}\frac{V_{odor}}{V_{odor}+V_{dil}+V_{const}} \end{array}$$

To simplify the calculation, we define the mixing factor K as the ratio $\frac{C_{final}}{C_{Vodor}}$. We obtain the following equation:

$$\begin{array}{ccc} K & = & \frac{V_{odor}}{V_{odor}+V_{dil}+V_{const}} \end{array}$$

To express the three different volumes delivered on period *T* as a function of the flow, the duty cycle and *T*, we integrate the flow with respect to time. We obtain the following equations:

$$\begin{array}{ccc} V_{odor} & = & \int_{0}^{\alpha T}F_{A}\left(t\right)dt=\alpha TF_{A}\;\\ V_{dil} & = & \int_{\alpha T}^{T}F_{A}\left(t\right)dt=T\left(1-\alpha \right)F_{A}\\ V_{const} & = & \int_{0}^{T}F_{B}\left(t\right)dt=TF_{B} \end{array}$$

Finally we obtain the expression of K as a function of the duty cycle and the flows:

$$\begin{array}{ccc} K(\alpha ) & = & \frac{F_{B}}{F_{B}+F_{A}}\alpha \end{array}$$

With this simplified theoretical model we obtain the function *K*(*α*) as a straight line with a slope of $\frac{F_{B}}{F_{B}+F_{A}}$.

### Measurement of mixing factor using gas chromatography

A measure of the mixing factor was assessed using gas chromatography. The molecule used was undiluted butanol. The airflow was the same as in the normal experimental paradigm: 0.6 L.min^−1^ for constant and odor airflows. The mixing factor (noted as *K*) was measured for twenty height values of duty cycle in random order. Each measure was conducted three times. A MATLAB program controlled the olfactometer and synchronized it with the chromatograph. Due to the valve delay, K increases strongly for extreme values of *α*. To improve these measures, the values of duty cycle are less spaced out for these values.

### Measurement of flow velocity rating

The opening and closing of the valve causes an oscillation of the airflow and consequently of air velocity. A measure of the oscillation’s magnitude was assessed using a pressure sensor (a Pitot tube) placed at the olfactometer’s outlet. The velocity was calculated with Bernoulli’s equation:

$$\begin{array}{ccc} u & = & \sqrt{\frac{2p}{\rho }} \end{array}$$

where *u* is flow velocity, *p* pressure and *ρ* air density. These measures were conducted for the constant airflow and the control/odor flow of 0.6, 0.9, 1.2 L.min^−1^ and for seven alpha values rated from 5% to 95% with step of 15%. For each measure, the signal was acquired during 30 s of stimulation and the magnitude was calculated with Fourier transform. The olfactometer used for this paper allowed a maximum flow rate of 4 L.min^−1^ but our air supply was restricted to 2.4 L.min^−1^.

### Behavioral study

#### Subjects

Twenty healthy subjects were included (mean age: 23.9 ± 3.2, 20 females) in this study. They were non-smokers, free from head colds and screened for any possible olfactory dysfunctions prior to the study. The study was reviewed and approved by the local ethics committee and declared to the relevant national authority in accordance with the Declaration of Helsinki on biomedical studies involving human subjects. Participants received information regarding the aim and procedures of the experiment, and gave their written informed consent to participate in the study. The protocol was approved by the Ethics Committee of Besançon University Hospital (ref 06/426).

Prior to the experiment, the olfactory functions of the participants were screened with a threshold test (Sniffin’ Sticks, Burghart Messtechnik, Wedel, Germany) [6], and subjects that obtained a score under 7 were discarded [16].

#### Odorant stimulation

Three odorants were used in this study: (1) pyridine (rotten fish odor) which is described as unpleasant in the literature; (2) butanol which is considered to have a neutral odor; (3) isoamyl acetate (IAA) (banana-like odor) which is usually described as a pleasant odor. Pyridine was diluted at 1%, butanol at 6% and isoamyl acetate at 20% in diethyl phtalate. Odors were diffused through a Teflon pipe (inside diameter: 1.6 mm) with its output placed at 5 cm of the two nostrils, which allows a birhinal stimulation. The constant airflow and the control/odor flow were set at 0.6 L.min^−1^ giving a total airflow of 1.2 L.min^−1^. Air velocity varied due to the opening and closing of the valves at rates from 0.06 to 0.4 m.s^−1^. These air velocity oscillations were under the threshold defined in the literature [17]. The humidity and temperature of airflow was identical to that of the room.

#### Procedure

Participants were comfortably seated in a well ventilated room where the light conditions and the ambient temperature were maintained at the same level for all subjects. A 15” screen was placed at about 50 cm of the subject’s head, and displayed instructions. The stimulation paradigm as well as data collection were computer-controlled with Eprime 2.0 (Psychology Software Tools, Pittsburgh, USA).

**Fig 5:**
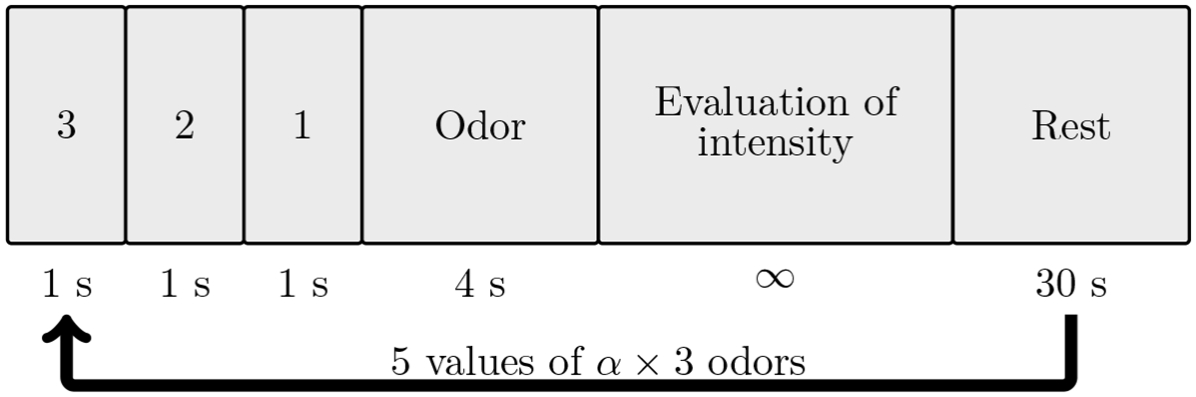
Schematic view of the experimental paradigm. After the countdown, odor is delivered and then, the participant rates its intensity. Each trial is followed by a rest period of 30 s.

**Fig 6:**
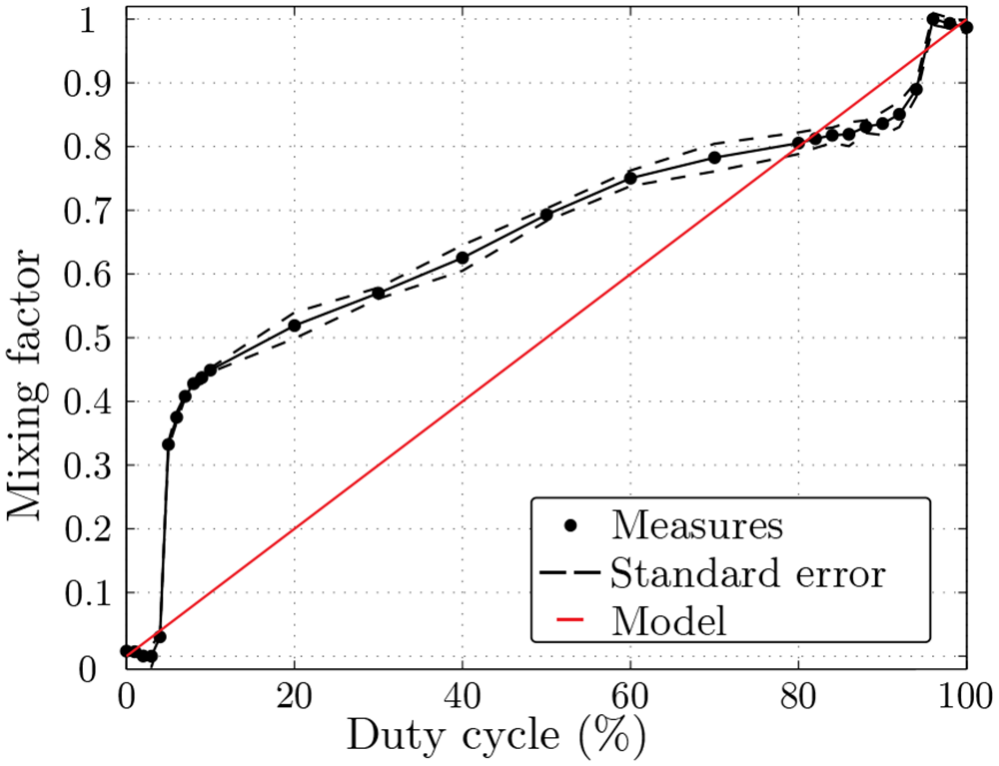
Gas chromatography measures of mixing factor *K* as a function of duty cycle *α*. Graph shows average and standard error of mixing factor *K* gas chromatography measures as a function of duty cycle *α*.

Each odor was diffused three times with five different mixing factors, bringing the total to fifteen stimulations per odorant. The mixing factors ranged from 33% to 100% in increments of 17%. A schematic view of the experimental procedure is depicted in Fig 5. The values of the corresponding duty cycles were determined with cubic interpolation of the curve in Fig 6. Each stimulation lasted 4 s, which corresponds to twenty switchings (i.e. opening and closing) of the valves. The stimulations were preceded by a countdown displayed on the screen, from 3 to 1 and lasting 3 s, at the end of which participants were asked to inhale [18]. Thanks to this method, the participants perceived the odor at the onset of the diffusion. After the stimulation, participants had to rate the intensity of the odor on a Likert scale displayed on the screen (from 0: not intense at all, to 9: extremely intense). Each try was always followed by a 30 s rest period, where there was no odorant stimulation. Lastly, the stimulations were randomly presented in three blocks for each odor, and the presentation order of these blocks was also randomized.

#### Data analysis

Statistical analysis of intensity ratings were performed using Statistica (StatSoft, Tulsa, USA). The three ratings per odorant and per mixing factor were averaged for each subject, so we obtained one value for each condition and each participant. These resulting data were analyzed using a one within factor analysis of variance (ANOVA) for repeated measures, for each odor, with the *α* as factor (five levels: *α* 5%, *α* 17%, *α* 46%, *α* 89% and *α* 100%). When there was a main effect of mixing factors, differences between levels were assessed using the Bonferroni post-hoc test.

## Results

### Gas chromatography

Contrary to the theoretical model described above, the concentration is not linear (Fig 6, S1 Table). The low values (from 0 to 4%) and the high values (from 96 to 100%) of *α* do not affect the mixing factor. Another consequence of this latency is the gap between the values of *α* inferior to 4% (*K* = 0) and 5% (*K* = 0.33). Therefore, the range for the mixing factor is limited to a range between 33% and 100% with the PWM method.

### Flow velocity variation

**Fig 7:**
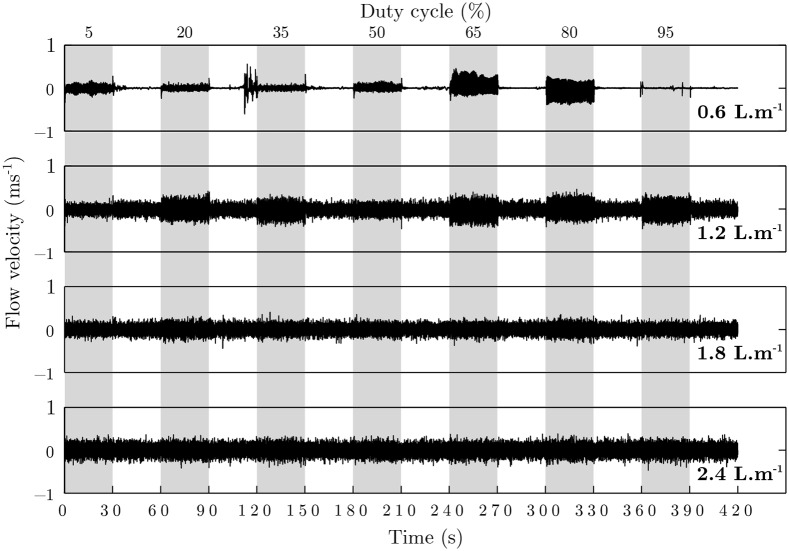
Flow velocity study. Measure of flow velocity during a stimulation (gray) and rest with airflow of 0.6, 1.2, 1.8 and 2.4 L.min^−1^ and with seven values of a duty cycle from 5 to 95% with step of 15%.

As shown by the curves of the air velocity variation, a rise in airflow modifies the amplitude of oscillations during both the stimulation and rest periods (Fig 7, S1 Data). Indeed, we observed that the natural oscillation of airflow velocity is weaker for a flow of 0.6 L.min^−1^ than for the highest flows. This is due to flow regime which is laminar at 0.6 L.min^−1^ (Re = 1.9 × 10^3^) and turbulent at 1.2 L.min^−1^ (Re = 5.1 × 10^3^) and over. Re is the Reynolds number. Is used to predict flow patterns (laminar or turbulent flow).

The flow rate modifies the air stream behavior during the stimulation. For airflows of 0.6 L.min^−1^, duty cycles of 65 and 80% clearly increase the amplitude of oscillations. At 1.2 L.min^−1^, we observed high oscillations for the majority of duty cycles: for 5 and 50%, the amplitude seems to be weaker. For the highest flow rates, the amplitude seems to decrease as the flow rate increases.

**Fig 8:**
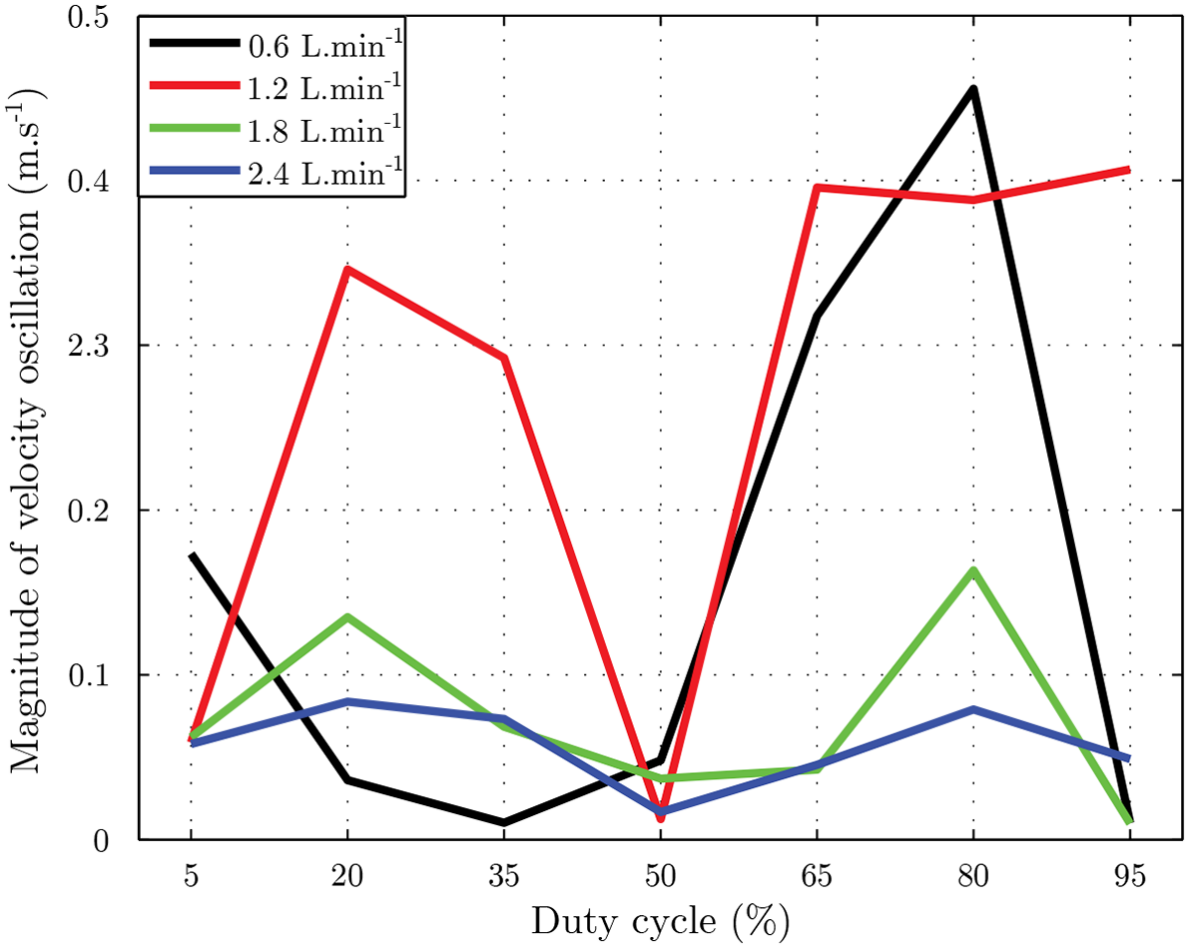
Flow velocity oscillations. Measure of air velocity oscillation caused by the use of PWM for airflow of 0.6, 1.2, 1.8 and 2.4 L.min^−1^ and with seven values of a duty cycle from 5 to 95% with step of 15%.

Fig 8 shows the peak to peak amplitude of oscillations due to PWM, calculated with Fourier transforms. These curves show that an increase in duty cycle from 5 to 20% amplifies the oscillation. From 20% to 50%, the curves go down and, at 50%, it moves up again to reach a peak at 80%. For the lowest flow rate, the first part of curve is different: the curve moves down to 5 from 35% and increases after this value. After 80%, the oscillations decrease except for the flow rate of 1.2 L.min^−1^.

The effect of duty cycle on oscillation can be explained by an asynchronous use of the valve. The effect can be different according to the flow rate due to the delay of the opening and closing valves, which depends on the air pressure. The curve of 0.6 L.min^−1^ is different from the others and thus an increase in flow rate causes a decrease in oscillation.

### Behavioral study

**Fig 9:**
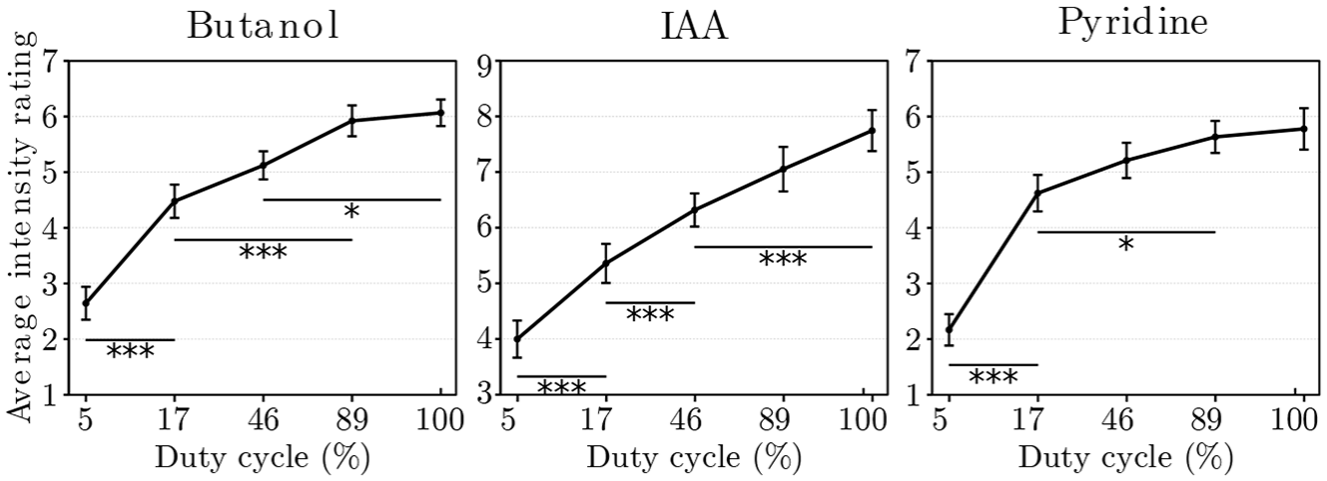
Averaged intensity ratings. Graph shows averaged intensity ratings (± standard error) of the five *α* for butanol, IAA and pyridine. *: *p* < 0.05, **: *p* < 0.01, ***: *p* < 0.001.

For the three odorants, the ANOVA revealed a significant main effect of the mixing factor. The results are *F*_(4,116)_ = 47.80 and *p* < 0.000 for the butanol, *F*_(4,116)_ = 42.54 and *p* < 0.000 for the IAA, and *F*_(4,116)_ = 46.47 and *p* < 0.000 for the pyridine. Bonferonni post-hoc tests revealed several differences between intensity ratings across *α* for butanol, IAA and pyridine. Theses results are detailed in Table 1, and the averaged intensity ratings for all odorants are depicted in Fig 9 (S2 Table). For *α* = 5%, 44 trials out of 297 (15%) were rated 0 by subjects.

**Table 1:** Post-hoc tests from ANOVA of intensity ratings. Differences of intensity ratings between *α* for the three odorants, assessed with p values from the Bonferonni test.

Odorant	Alpha	*α* 5%	*α* 17%	*α* 46%	*α* 89%
Butanol	*α* 5%				
	*α* 17%	0.000000			
	*α* 46%	0.000000	0.261513		
	*α* 89%	0.000000	0.000017	0.060478	
	*α* 100%	0.000000	0.000002	0.012785	1.000000
IAA	*α* 5%				
	*α* 17%	0.000400			
	*α* 46%	0.000000	0.031580		
	*α* 89%	0.000000	0.000005	0.230537	
	*α* 100%	0.000000	0.000000	0.000176	0.314728
Pyridine	*α* 5%				
	*α* 17%	0.000000			
	*α* 46%	0.000000	0.569313		
	*α* 89%	0.000000	0.012775	1.000000	
	*α* 100%	0.000000	0.002553	0.668005	1.000000

## Conclusion and Discussion

The theoretical model offered as a way of determining the relationship between duty cycle and concentration helps to understand the principle of PWM applied to odor presentation. The data chromatography shows the limits of the model. Although the curve is not linear, the increase in duty cycle induces an increase in concentration (except for extreme values of duty cycle). A characterization is also necessary to determine the relation between the duty cycle and the mixing factor.

With most dynamic olfactometers, the opening and closing of the valves create a modification of flow rate. With PWM, the modification of flow rate causes an oscillation of the air velocity during a stimulation. With the present olfactometer, the variation in velocity rates from 0.01 to 0.45 m.s^−1^ depending on flow rate and duty cycle. Clarke and Jones [17] showed that the minimum consciously detectable velocity range at the nasal vestibule is 6.5 m.s^−1^. Thus, with our olfactometer, the oscillations of air velocity could not be detected by the participants. However, our study does not assess the sensibility of human subjects to theses oscillations of air velocity. Further studies could therefore be very useful to determine whether theses variations are able to modulate olfactory perception. Indeed, airflow oscillations could produce trigeminal sensations that are known to play a key role in human nasal chemoreception [19] and in the unconscious perception of odors [20]. The changeable nature of air stream’s behavior from one olfactometer to another is difficult to predict. So each system should be individually characterized to verify that variations are acceptable, and do not interfere with olfactory perception. In our case, studying the effect of velocity range on olfactory and trigeminal perception will be taken into account in our further studies of PWM applied to odorant stimulations using our olfactometer.

Our behavioral results suggest that the mixing factor (and thus the duty cycle) modulates the intensity ratings. Indeed, stimulations with low duty cycles induce significantly lower intensity ratings than stimulations with larger height duty cycles. These results are similar to those of other studies that modify odorant concentrations [12, 13], and are congruent with those from our gas chromatography study.

However, although our data show a significant difference between *α* 5% and other *α* for the three tested odorants, all *α* are not systematically different from each other (Table 1). Moreover, the differences between the *α* are not the same according to the odorant. With pyridine for example, the higher *α* values are not significantly different from each other and induce intensity ratings between 5 and 6. Thus, duty cycles have to be adapted for each odorant to allow a more accurate modulation of perceived intensity.

Nevertheless, gas chromatography and psychophysical data show that duty cycles modulate odorant concentrations and consequently intensity ratings in human subjects. Thus, PWM applied to olfactory stimulation could be used in many experiments where quick and effective variations of concentration are needed. We can imagine paradigms with quick successions of different odors with varied concentrations, or the same odorant with a series of different concentrations. Thanks to its easy computer control which allows automation, we can also imagine fully automated paradigms, like olfactory threshold tests. PWM could be a solution to create a continuous gradient of an odor that would be very useful in immersive virtual experiences [21]. Lastly, it can also be used to quickly adjust odor intensity for each subject. Indeed, some studies need an individual intensity adjustment of odors, and PWM could offer an quick and easy way to do it [21].

**Fig 10:**
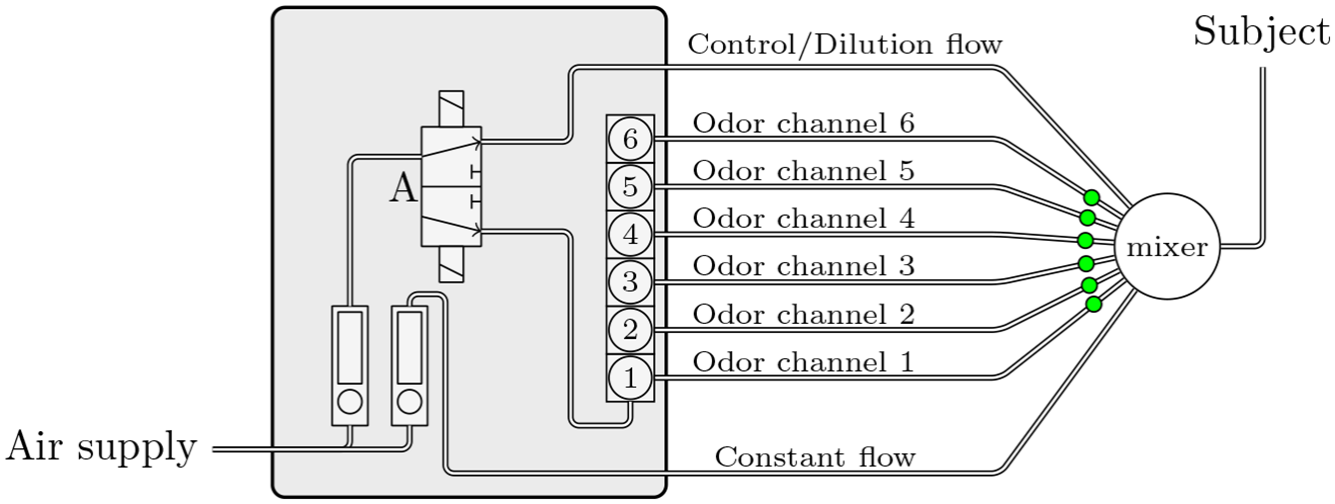
Setting up of a fast three-way valve in case the solenoid valves are too slow. In case the solenoid valves are too slow, the pneumatic system can be improved by adding a fast three-way valve. This valve alternatively supplies the control/dilution channels and odor channels.

PWM is a versatile method which allows computer-controlled tuning of odor concentration and can be implemented in most olfactometers without major modification and at a low cost. For the olfactometers using solenoid valves that are too slow, a fast three-way valve can be added. In this configuration, the three-way valve noted A in Fig 10 chops the airflow: it directs the flow directly towards the mixer or supplies the valves 1 to 6. Valves 1 to 6 are only needed to select the odor channel.

In case a larger range of concentration is needed, another dilution of odor can be used: therefore, a supplementary odor channel would be used. In this case, it would be possible to use a very large range of concentrations with just two or three dilutions.

## Supporting Information

S1 Table Gas chromatography data. (ODS)

S1 Data RAW data presented in Fig 7. (MAT)

S2 Table Subjective ratings of odor intensities. (ODS)
